# Mechanisms Underlying Resistance to FLT3 Inhibitors in Acute Myeloid Leukemia

**DOI:** 10.3390/biomedicines8080245

**Published:** 2020-07-24

**Authors:** Motoki Eguchi, Yosuke Minami, Ayumi Kuzume, SungGi Chi

**Affiliations:** 1 Department of Hematology, National Cancer Center Hospital East, Kashiwa 277-8577, Japan; guchimomo.may.1st@gmail.com (M.E.); akuzume@east.ncc.go.jp (A.K.); schi@east.ncc.go.jp (S.C.); 2 Division of Hematology/Oncology, Department of Internal Medicine, Kameda Medical Center, Kamogawa 296-8602, Japan

**Keywords:** acute myeloid leukemia (AML), FMS-like tyrosine kinase 3 (FLT3), quizartinib, gilteritinib

## Abstract

FLT3-ITD and FLT3-TKD mutations were observed in approximately 20 and 10% of acute myeloid leukemia (AML) cases, respectively. FLT3 inhibitors such as midostaurin, gilteritinib and quizartinib show excellent response rates in patients with FLT3-mutated AML, but its duration of response may not be sufficient yet. The majority of cases gain secondary resistance either by on-target and off-target abnormalities. On-target mutations (i.e., FLT3-TKD) such as D835Y keep the TK domain in its active form, abrogating pharmacodynamics of type II FLT3 inhibitors (e.g., midostaurin and quizartinib). Second generation type I inhibitors such as gilteritinib are consistently active against FLT3-TKD as well as FLT3-ITD. However, a “gatekeeper” mutation F691L shows universal resistance to all currently available FLT3 inhibitors. Off-target abnormalities are consisted with a variety of somatic mutations such as *NRAS*, *AXL* and *PIM1* that bypass or reinforce FLT3 signaling. Off-target mutations can occur just in the primary FLT3-mutated clone or be gained by the evolution of other clones. A small number of cases show primary resistance by an FL-dependent, FGF2-dependent, and stromal CYP3A4-mediated manner. To overcome these mechanisms, the development of novel agents such as covalently-coupling FLT3 inhibitor FF-10101 and the investigation of combination therapy with different class agents are now ongoing. Along with novel agents, gene sequencing may improve clinical approaches by detecting additional targetable mutations and determining individual patterns of clonal evolution.

## 1. Introduction

FMS-like tyrosine kinase 3 (FLT3) is classified as a type 3 receptor tyrosine kinase, along with KIT, FMS, and PDGFR [1,2,3]. FLT3 is composed of an extracellular region consisting of five immunoglobulin-like domains, and an intracellular region consisting of a juxtamembrane (JM) domain, two tyrosine kinase (TK) domains, and a C-terminal domain. FLT3 is expressed in normal hematopoietic stem cells and progenitor cells, and is dimerized upon binding with either membrane-bound or soluble FLT3 ligands (FLs) produced by bone marrow stromal cells, which subsequently causes the phosphorylation and activation of tyrosine residues in the activation-loop (A-loop) [4,5]. Phosphorylated FLT3 activates multiple intracellular signaling pathways involved in the survival, proliferation, and differentiation of hematopoietic stem cells, such as RAS/MAPK, PI3K/Akt/mTOR, and JAK/STAT5 [6,7,8,9]. Since FLT3 is frequently expressed in leukemic cells, FL stimulation induces proliferation and inhibits apoptosis in these cells [10,11]. In 1996, an internal tandem duplication in the JM domain-encoding region of *FLT3* (FLT3-ITD) was identified in acute myeloid leukemia (AML) cells [12]. Thereafter, several types of mutations, including point mutations, deletions, and insertions have been detected around the D835 residue in the TK domain (FLT3-TKD) [13]. FLT3-ITD and FLT3-TKD mutations were observed in approximately 20 and 10% of AML cases, respectively [14,15,16]. Although both FLT3-ITD and FLT3-TKD are gain-of-function mutations, the upregulation of STAT5 was only observed in FLT3-ITD cell lines (32D/ITD) [17]. STAT5 positively regulated Pim-1, which eventually activated mTOR and Mcl-1, which consequently conferred resistance to Akt inhibition in FLT-ITD cell lines [18]. An experiment using transgenic mice with FLT3-ITD-positive hematopoietic stem cells revealed the clear promoting effects of nuclear factors in activated T-cells (NFATC1), a family of inflammatory transcriptional factors, on FLT3-ITD-driven precursor cell expansion and resistance to FLT3 inhibitors [19]. Recent studies suggest that circulating MYBL2, encoded by the cell-cycle checkpoint gene *MYBL2*, is detected in AML patients with FLT3-ITD mutations and is closely related to mutant FLT3 expression as well as to tumor cell activity [20]. Unlike FLT3-ITD consistently upregulating JAK/STAT signaling, FLT3-TKD enhance SHP1 and SHP2 activity that negatively regulate JAK signaling [21,22]. This may at least partially explain why FLT3-ITD showed more potent myeloproliferative advantages than those of FLT3-TKD in a mouse model [23,24]. The dual mutation of FLT3-ITD and -TKD (FLT3-ITD-TKD) has been found in a small population. A recent study showed that FLT3-ITD-TKD has the ability to activate STAT5, resulting in Bcl-x and RAD51 upregulation that accounts for drug resistance [25]. Since FLT3 mutations are frequently detected in AML and are associated with poor prognosis, this gene is considered a promising molecular target for AML [26,27]. It has been 20 years since abnormalities in the *FLT3* were first discovered, and the application of FLT3 inhibitors in clinical settings in Japan, Europe, and the United States has resulted in a paradigm shift in the treatment of FLT3-mutated AML. However, resistance to FLT3 inhibitors has also been reported concomitantly. Mechanisms of the resistance and strategies to overcome it have been vigorously studied and ever-reviewed [28,29,30]. Along with the comprehensive understanding of pathologic FLT3 signaling and the acquired alterations responsible for drug-resistance, non-FLT3 abnormalities that may be closely associated with leukemic clone evolution are revealing its importance, suggesting new approaches. In this review, we summarize our current understanding of resistance to FLT3 inhibitors and discuss the strategies for overcoming this issue.

## 2. Prognostic Impact of FLT3 Mutations

FLT3-ITD mutation has been recognized as one of the major adverse prognostic factors with nearly twice the increase in hazard ratio [31]. As mentioned in the European LeukemiaNet (ELN) recommendations [27], high allelic burden (generally indicating 50% or more) of FLT3-ITD (FLT3-ITD^high^) is consistently associated with worse prognosis [32,33,34]. On the other hand, the low allelic frequency of FLT3-ITD (FLT3-ITD^low^) concomitant with NPM1 mutation possibly leads to favorable prognosis [35], though it has been fraught with controversy [36,37,38]. FLT3-ITD^high^ with wild type NPM1 and FLT3-ITD^low^ with mutated NPM1 are classified as intermediate-risk [27]. Unlike FLT3-ITD, the prognostic significance of FLT3-TKD has not been determined [32,39]. With the development of potent FLT3 inhibitors, better clinical outcomes would be expected, especially in patients with FLT3-ITD^high^. Indeed, previously untreated FLT3-ITD^high^ patients who received intensive chemotherapy with sorafenib, a FLT3 inhibitor, showed no significant but seemingly better relapse-free and overall survival than those with FLT3-ITD^low^ AML [34]. It is not fully known if the FLT3 allelic burden affects the properties in acquiring resistance to FLT3 inhibitors. However, given a certain somatic mutation will belong to a single clone, a larger proportion of mutant FLT3 allele may link to less divergent leukemic clones and vice versa, which theoretically affect drug sensitivity, relapse rates and eventually survival rates. Zhang and his colleagues graphically displayed the clonal evolutions of two individual cases; one for a single clone with a high frequency of FLT3-TKD that later relapsed with an additional mutation within the same clone and the other for complex clones not associated with first-detected FLT3-ITD mutation with low frequency [40]. The prognostic impact of FLT3 mutations and its allele frequency possibly be changed in the era of FLT3 inhibitors.

## 3. Classification of FLT3 Inhibitors by Its Pharmacodynamics

As first-generation FLT3 inhibitors, existing TK inhibitors such as tandutinib (CT53518), lestaurtinib (CEP-701), sunitinib (SU11248), midostaurin (PKC412), and sorafenib (BAY 43-9006), which can effectively inhibit FLT3 kinase have been studied [41,42,43,44,45]. Thereafter, the compounds with higher selectivity and inhibitory activity were identified. Gilteritinib (ASP2215), quizartinib (AC220), and crenolanib (CP868596) were developed as second-generation FLT3 inhibitors [46,47,48,49,50]. These FLT3 inhibitors are roughly classified into two types (i.e., type I and type II) based on their binding mode to FLT3 molecules. The conformation of the three amino acid residues Asp–Phe–Gly (DFG) in the A-loop of the FLT3 molecule is altered in accordance with the phosphorylation status of the tyrosine residue, which leads to the formation of an active DFG-in conformation or an inactive DFG-out conformation [51,52,53]. Type I inhibitors bind to the ATP-binding site and its vicinity, and subsequently bind with molecules in both DFG-in and DFG-out conformations. Since the molecular homology of various TKs is high and the ATP-binding sites are highly conserved among kinases, type I inhibitors are often less selective. In contrast, type II inhibitors bind to the target kinase by utilizing the hydrophobic space that appears in the proximity of the ATP-binding site in the DFG-out conformation. Since the hydrophobic space in this structure varies significantly between various kinases, type II inhibitors are expected to be more selective than type I inhibitors and are unable to inhibit activated kinases in the DFG-in conformation. Midostaurin, gilteritinib, and crenolanib are type I inhibitors, while quizartinib and sorafenib are type II inhibitors [54]. FLT3-TKD maintains a constant DFG-in conformation owing to alterations in the TK domain, whereas FLT3-ITD can exist in both active DFG-in conformation and inactive DFG-out conformation. Therefore, while type I inhibitors inhibit both FLT3-TKD and FLT3-ITD, type II inhibitors only inhibit FLT-ITD owing to the differences in binding properties, with a few exceptions in first-generation agents (e.g., midosutaurin and sunitinib). For example, TK domain-altering D835 point mutations confer resistance to a type II second-generation inhibitor quizartinib, but not to type I gilteritinib and crenolanib [55]. However, a “gatekeeper” mutation F691L shows universal resistance to all the currently available FLT3 inhibitors [47,49,56,57,58,59]. The characteristics of the FLT3 inhibitors are summarized in Table 1.

## 4. Current Clinical Role of FLT3 Inhibitors

Among a number of tyrosine kinase inhibitors active against pathologic FLT3 signaling, gilteritinib and midostaurin are now available for the treatment of FLT3-mutated AML in most developed countries. Quizartinib is currently available only in Japan. Stone and his colleagues reported a randomized phase 3 trail, RATIFY, where midostaurin or placebo were added to standard therapy in patients with newly diagnosed FLT3-mutated AML [73]. The midosutaurin group showed longer survival (hazards ration (HR) 0.78) and improved event-free interval (HR 0.78) than the counterpart. Recently, the combination of midostaurin and standard therapy followed by midostaurin maintenance also showed better outcomes compared with historical controls (HR 0.58 in event-free survival) [74]. Efficacy of single-agent gilteritinib for relapsed/refractory FLT3-mutated AML was proved in a randomized phase 3 trial, ADMIRAL [75]. The median overall survival was significantly longer in the gilteritinib group than the conventional chemotherapy group (9.3 months vs. 5.6 months), with a higher percentage of patients who underwent allo-stem cell transplantation (SCT) (26% vs. 15%). However, the median event-free interval was less than 3 months. Similarly, the phase 3 QuANTUM-R trial showed the superiority of single-agent quizartinib over salvage chemotherapy in the same situation (HR 0.76 in overall survival) [76]. Quizartinib has also been tested in the first-line setting and showed activity in a phase 1 trial [77]. In addition to the approved drugs mentioned above, other FLT3 inhibitors also have displayed clinical benefits. Published trials and their primary results are summarized in Table 2. Sorafenib, already approved for renal cell cancer, thyroid cancer and hepatocellular carcinoma, were evaluated in either in a first-line and salvage situation combined with chemotherapy and HMAs (hypomethylating agents), showing promising results [78,79,80,81,82,83,84]. A novel second-generation FLT3 inhibitor crenolanib has shown possible benefits in combination with conventional chemotherapy, in either first-line and salvage treatment [85,86,87]. Lestaurtinib, however, failed to display clinical benefit when administered as maintenance therapy following standard treatment [88,89].

## 5. Mechanisms of Resistance to FLT3 Inhibitors

### 5.1. Primary Resistance

**Figure 1:**
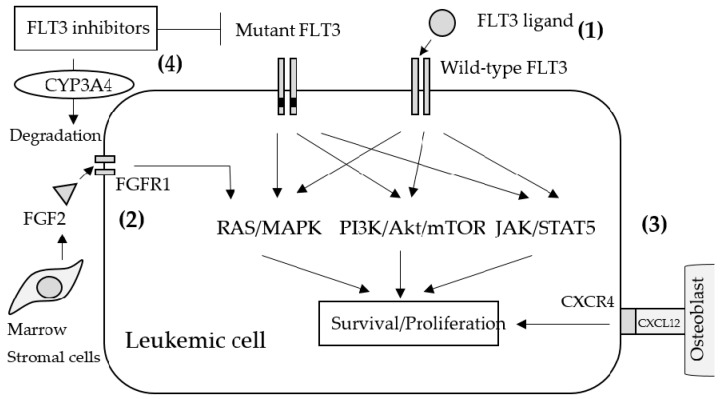
Schematic mechanisms of primary resistance to FLT3 inhibitors. (1) Wild-type FLT3s are a little sensitive to FLT3 inhibitors and allow downstream signaling by binding with FLT3 ligands. (2) FGF2 secreted from bone marrow stromal cells activates FGFR1 on leukemic cells which leads to MAPK activation. (3) Cell adhesion to the microenvironment may also help leukemic proliferation. Antagonizing CXCR4 that binds to CXCL12 on osteoblasts resulted in attenuated leukemia progression. (4) Upregulating CYP3A4 leads to the rapid inactivation of FLT3 inhibitors.

Resistance to FLT3 inhibitors can be classified as primary resistance (innate resistance) and secondary resistance (acquired resistance). In primary resistance, the effect of FLT3 inhibitors are prevented during the initial administration in an FL-dependent, FGF2-dependent, and stromal CYP3A4-mediated manner as well as by the activation of other signaling pathways (Figure 1). Most FLT3-mutant AML cells also express wild-type (WT) FLT3 concomitantly. Since WT-FLT3 is sensitive to FL and is affected negligibly by FLT3 inhibitors, FL secretion in the bone marrow microenvironment leads to the activation of the FLT3/MAPK pathway and provides survival signals to AML cells during induction and consolidation therapy. Indeed, certain studies have demonstrated that the co-existence of WT-FLT3 attenuated the anti-tumor effects of FLT3 inhibitors on FLT3-mutated AML cells in vitro and in vivo [88,90,91]. In addition to FL, other cytokines, growth factors, and soluble proteins from the bone marrow microenvironment have been studied with respect to their resistance against quizartinib. For example, fibroblast growth factor 2 (FGF2) induces resistance by activating FGFR1 and inducing downstream MAPK signaling. FGF2 expression in bone marrow stromal cells increased in patients with FLT3-ITD-positive AML treated with quizartinib and was maximized prior to clinical relapse and the induction of resistance mutations [92]. CXCL12, a chemokine expressed by osteoblasts in the bone marrow, is a ligand of CXCR4 expressed by hematopoietic stem cells as well as AML cells. Certain reports revealed that the CXCR4 antagonist plerixafor (AMD 3100) selectively reduced the proliferation of FLT3-ITD AML blasts and increased the sensitivity of FLT3-mutated leukemic cells to the apoptogenic effects of FLT3 inhibitors [93,94]; therefore, the activation of the CXCL12/CXCR4 axis may also induce resistance to FLT3 inhibitors in AML cells. The inactivation of TKIs by CYP3A4 is well established. In particular, hepatic CYP3A4 inactivates all TKIs, including FLT3 inhibitors. Additionally, the expression of CYP3A4 in bone marrow stromal cells attenuated the activity of three different FLT3 inhibitors (sorafenib, quizartinib, and gilteritinib) in FLT3-ITD-positive AML [95].

### 5.2. Secondary Resistance Due to Additional FLT3 Mutations (on-Target Resistance)

**Figure 2:**
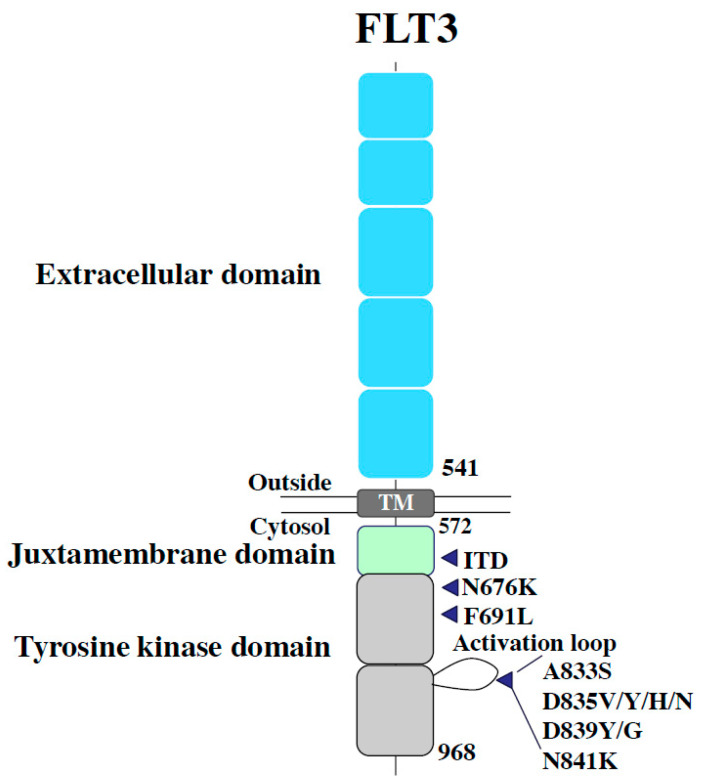
Additional FLT3 tyrosine kinase domain mutations responsible for secondary on-target resistance. These mutations keep the TK domain in active FDG-in form, not allowing the type II inhibitors to bind there. Mutations in a “gate-keeping” residue F691 shows the universal resistance to both type I and II inhibitors.

Secondary resistance negates the effects of FLT3 inhibitors via the abnormalities acquired by FLT3 inhibition, such as additional mutations in FLT3 (“on-target” resistance) and defective factors apart from FLT3 (“off-target” resistance). Several genetic mutations associated with FLT3 inhibitor resistance have been reported in clinical trials on FLT3 inhibitors. As mentioned earlier, since type II inhibitors originally have no affinity for FLT3-TKD, additional mutations in the TK domain can confer resistance via the elimination of the inhibitory effect on FLT3-ITD. In cases of recurrence after quizartinib treatment in patients with FLT3-ITD-positive AML, secondary mutations at D835 and Y842 residues as well as at the commonly known “gatekeeper residue” F691 in the kinase domain have been reported (Figure 1) [96]. In vitro, Ba/F3 cells expressing FLT3-ITD and one additional TKD mutation, detected in patients with clinical resistance (+D835Y, +D835V, +Y842C, +Y842H, or +F691L), exhibited resistance to the growth inhibitory effect and dephosphorylation activity of quizartinib. These resistance mutations in the A-loop were also observed in patients treated with sorafenib, another type II inhibitor. Furthermore, during the treatment with gilteritinib and crenolanib (a type I inhibitor), the additional appearance of FLT3-TKD mutations in patients with resistance was infrequent, although the appearance of F691L, a gatekeeper mutation, was observed (Figure 2).

Although the FLT3-F837K and FLT-C35S mutations occurred after the gilteritinib treatment in one patient each, both were considered silent mutations as these did not induce self-proliferation in Ba/F3 cells^40^. Among the 50 resistant patients treated with crenolanib, five FLT3 (D200N, K429F, Y572C, L601F, and F691L) mutations were observed in six patients; the D200N and L601F mutations did not result in leukemia [40]. Since the frequency of the acquired mutations in FLT3-ITD in patients with clinical resistance to quizartinib, a type II inhibitor, was 50% or less, other resistance mechanisms are also anticipated. In four out of eight patients treated with quizartinib, one or more resistance mutations were observed in the TK domain [96]. In addition to FLT3-ITD alleles, mutations in the TK domain of the original FLT3 allele were detected in seven individuals. Notably, the patients exhibited different frequencies of mutations between the original FLT3 allele and the FLT-ITD allele. In this study, the AML cells collected from one quizartinib-resistant patient did not acquire resistance mutations in either the original FLT3 allele or the FLT3-ITD allele. No mutations were detected in the genes apart from *FLT3*, although the existence of off-target resistance mechanisms was considered in this patient. These findings suggest the existence of a polyclonal resistance mechanism in patients with AML that relapses after quizartinib treatment.

### 5.3. Secondary Resistance Due to Non-FLT3 Abnormalities (off-Target Resistance)

**Figure 3:**
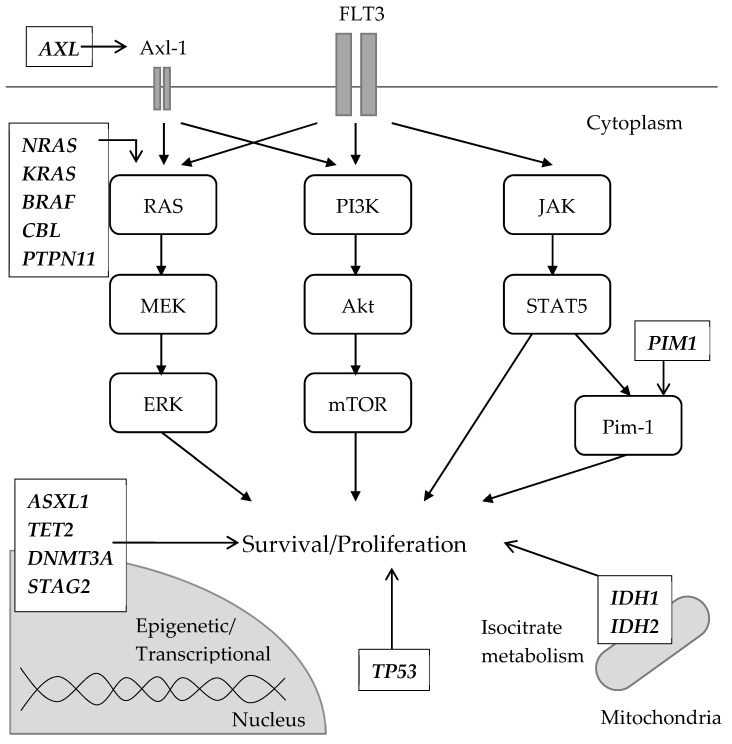
Schematic description of genetic abnormalities (mutations or upregulation) associated with secondary off-target resistance to FLT3 inhibitors. Mutations involved in the RAS/MAPK pathway were reported. *NRAS* mutation is the most common among them. Axl-1, coded by the *AXL* gene, is a receptor tyrosine kinase that leads to the activation of RAS/MARK and PI3K/Akt/mTOR pathway. The upregulation of the *AXL* gene was observed in midostaurin-resistant AML cell lines. Pim-1 is part of the downstream signaling of STAT5, contributing cell survival and proliferation as well as cell migration. A lestaurtinib-resistant AML cell line showed the overexpression of Pim-1. Other gene mutations commonly seen in AML regardless of FLT3 status were also detected. Although a direct relationship with FLT3 signaling was not suggested, these mutations have an essential role in maintaining leukemic clones by modulating epigenetic/transcriptional regulations (e.g., *ASXL1*, *TET2*, *DNMT3A* and *STAG2*), altering the metabolism of the citrate acid cycle (e.g., *IDH1* and *IDH2*) and preventing apoptosis (e.g., *TP53*).

Resistant clones formed after treatment with gilteritinib and crenolanib, and a type I inhibitor that exerts an inhibitory effect on FLT3-TKD, have characteristics that are different from those observed after treatment with type II inhibitors. In a comparative genetic analysis before and after relapse in patients treated with gilteritinib, several distinct patterns of clonal selection were observed during the treatment period with gilteritinib [97]. In five out of 41 (12.2%) gilteritinib-resistant patients, FLT3 mutations were not observed in AML cells after the gilteritinib treatment; however, mutations in the RAS/MAPK pathway were present in all of the patients. These results suggest that mutant FLT3-negative clones acquire mutations in the RAS/MAPK pathway and expand as resistant clones. In 36 other patients, the resistant clones contained the original FLT3 mutation, and five of them acquired an F691L TKD mutation in addition to the original FLT3 mutation. In 10 out of the 36 patients with the original FLT3 mutation, additional mutations in the RAS/MAPK pathway, such as *NRAS, KRAS, PTPN11, CBL,* and *BRAF* mutations, were acquired. Of note, the mutations in the RAS/MAPK pathway and FLT-F691L mutations were mutually exclusive. In vitro experiments conducted in MOLM-14, an AML cell line with FLT3-ITD, where either mutant RAS or FLT3-F691L was transduced into the parental cells and gilteritinib was administered at low/high-dose (25 and 250 nmol/L), suggested that the RAS-mutant clones were more likely selected by the high concentration of the inhibitor, besides the FLT3-F691L which was more likely to be selected by a low one. Similar to RAS mutations [97,98], the activation of Axl-1, a member of the TAM family of receptor TKs, may also contribute to FLT3-resistance by constantly activating the RAS/MAPK and PI3K/Akt/mTOR pathway. Axl-1 was observed to be highly phosphorylated in midosutaurin-resistant AML cell lines and its resistance was diminished by the Axl-1 inhibition in vitro [99]. In another experiment, patient-derived AML cells with FLT3-ITD were co-cultured with stromal cells and treated with quizartinib [100]. The surviving cells underwent STAT5 activation, which consequently upregulated AXL, which was further enhanced by the hypoxic environment. Conversely, in patients eliciting poor response to crenolanib, several abnormalities have been observed in the loci encoding epigenetic regulators and granulocyte transcription factors, as well as in the cohesin complex. In particular, *NRAS*, *STAG2*, *CEBPA*, *ASXL1*, and *IDH2* mutations were observed in FLT3-WT clones [40]. These findings suggest that the clones escaped and expanded during crenolanib therapy. However, *TET2*, *IDH1*, and *TP53* mutations occurred simultaneously in FLT3-mutated clones during crenolanib treatment. These results suggest that the off-target resistance mechanism is more frequent when using type I inhibitors, such as gilteritinib or crenolanib, than type II inhibitors. Besides, IDH1 inhibitor ivosidenib [101] and IDH2 inhibitor enasidenib [102,103], both approved by the FDA, are active against IDH1/2-mutant relapsed/refractory AML, though the significance of co-existing FLT3 mutations is not fully understood. In addition, the upregulation of the PI3K/AKT/mTOR pathway in resistant cell lines treated with sorafenib has also been reported [104]. Pim-1 is a proto-oncogene originally detected in hematopoietic cells that functions downstream of STAT5 [105]. Its overexpression induced resistance to lestaurtinib in BaF3/ITD cells and in samples collected from FLT3-ITD-positive patients [106]. Additionally, Pim kinase overexpression has been observed in the samples collected post sorafenib administration in patients with FLT3-ITD-positive AML compared to the levels observed in the samples collected before administration [107]. Pim-1 was associated with an increased expression of anti-apoptosis proteins, such as Bcl-2, BCL-XL, and MCL-1, in FLT3 inhibitor-resistant cases [25,108,109,110]. In particular, the observed resistance may be partly induced by Pim-1. Off-target abnormalities along with FLT3 signaling are schematically summarized in Figure 3.

## 6. Strategies to Overcome Resistance to FLT3 Inhibitors

### 6.1. Development of Novel Agents

Previous reports suggest that on-target resistance tends to occur in patients after type II inhibitor treatment, while off-target resistance is likely to occur after type I inhibitor treatment. Since these reports are currently limited to patients recruited during clinical trials, for a better understanding of the mechanism underlying the resistance to each FLT3 inhibitor, it is necessary to determine the characteristics of patients with resistance in real-word settings. In addition, to counter the gatekeeper mutation (F691L) in *FLT3*, which confers resistance to all existing FLT3 inhibitors, it is necessary to develop a novel FLT3 inhibitor. As described above, while type I inhibitors can also inhibit FLT3-TKD, they exhibit low selectivity, whereas although type II inhibitors cannot inhibit FLT3-TKD, they exhibit high selectivity. FLT3-TKD inhibitory activity and FLT3 selectivity share a trade-off relationship. To resolve these issues, a novel FLT inhibitor known as FF-10101 was designed, which would form covalent bonds with the C695 residues of FLT3. The creation of covalent bonds by FF-10101 enables the selective and irreversible inhibition of FLT3 in either the active or the inactive form [111]. Furthermore, the unique binding method of FF-10101 exerts wide inhibitory action against various *FLT3* mutations, including F691L. Currently, phase 1/2 trials are underway to evaluate its safety, tolerability, pharmacokinetics, and efficacy against recurrent refractory AML (NCT03194685). In addition, several agents that may overcome or prevent resistance are currently under investigation. A pan-PIM/FLT3 inhibitor SEL24 [112], a type II FLT3 inhibitor MZH29 [113], a MERTK/FLT3 inhibitor MRX-2843 [114], a BCR-ABL inhibitor ponatinib [115], and a multiple tyrosine kinase inhibitor cabozantinib [116] have exhibited anti-tumor activity in cases with FLT3-TKD, including those with the F691 pointmutation.

### 6.2. Combination with Different Class Agents

Existing FLT3 inhibitors are now being tested in combination with HMAs, standard chemotherapy, bortezomib (proteasome inhibitor), atezolizumab (anti-PD-L1 antibody), venetoclax (BCL-2 inhibitor), milademetan (MDM2 inhibitor) and homoharringtonine (STAT inhibitor). Ongoing trails of combination strategy are summarized in Table 3 Preclinically, FLT3 ligand-mediated resistance was attenuated by the dual inhibition of AKT/FLT3 in vivo [117]. The combination of the MEK and FLT3 inhibitors as well as the dual inhibition of MEK/FLT3 proved to be effective against resistance-conferring FLT3 mutations in in vivo and in vitro mutations [97,118]. The sensitization of FLT3 inhibitors can serve as an alternate strategy. Proteasome inhibitors, arsenic trioxide (ATO), and a CDK4/6 inhibitor palbociclib downregulated FLT3 molecules in FLT3-ITD AML cells by promoting cytotoxic autophagy, inhibiting the expression of FLT3 RNAs, and dysregulating the transcription of *FLT3* and *PIM1*, respectively [119,120,121]. The inactivation of *ATM* or its downstream effector G6PD also induced synthetic lethality along with *FLT3* inhibition by enhancing mitochondrial oxidative stress, which eventually resulted in tumor apoptosis [122].

### 6.3. Genetic Mutation Analysis

As described, the presence or absence of mutations in *FLT3* has become an important determinant of the treatment methods in AML. Currently, a companion diagnostic tool LeukoStratCDx (Invivoscribe, Inc., San Diego, CA, USA) is widely used for the clinical use of FLT3 inhibitors in Japan, the United States, and Europe, among others. However, LeukoStratCDx is only able to detect D835 and I836 mutations and cannot detect any other FLT-TKD mutations, including F691L. Therefore, the instrument might incorrectly analyze the condition in patients with FLT3-TKD that is potentially treatable by FLT3 inhibitors. Although intensive chemotherapy has ensured substantial clinical benefit in AML patients, several patients eventually require targeted therapy, particularly young patients. In addition, *CEBPA* and *NPM1*, and recently *TP53*, *ASXL1* and *RUNX1*, have been determined to be important markers prognosis [27,31,123], transplant eligibility, and treatment strategy. Even after FLT3-ITD/TKD becomes undetectable in remission, the expression of persistently mutated genes such as *DNMT3A*, *TET2*, *SRSF2*, and *ASXL1* continues to be associated with high relapse rates and poor prognosis [124]. Although the negative prognostic impact of FLT3-ITD might be, at least partially, attenuated by upfront haploidentical stem cell transplantation (haplo-SCT) [125], FLT3 inhibitors remain one of the useful choices for treating the majority of FLT3-mutated AML patients, especially elderly and/or unfit people. To overcome resistance to FLT3 inhibitors, mutation analyses in patients with resistance to FLT3 inhibitors are required to identify the genetic abnormalities that contribute to drug-resistance and determine additional therapeutic targets. Genome-wide analysis using the CRISPR-Cas9 single-guide RNA (sgRNA) library, a vector-mediated technique for the knockdown of particular genes, revealed that the loss of *SPRY3* and *GSK3* confers resistance to quizartinib by inducing the reactivation of the FGF/RAS/ERK pathway and Wnt signaling [126]. Likewise, in addition to FLT3 mutations, it is necessary to comprehensively evaluate various genetic abnormalities; comprehensive mutation testing by next-generation sequencing (NGS) is expected to enable this. Accordingly, we analyzed the cancer-related genetic abnormalities (i.e., in an NGS panel) in patients with AML who were ineligible for intensive chemotherapy or developed recurrent/refractory cancer after initial therapy (Foundation One Heme; we planned HM-SCREEN-JAPAN, an observational study that analyzes and evaluates the relationship between prognosis by F1H). The primary goal of this project is the development of F1H and the promotion of targeted therapy for AML [127].

## 7. Conclusions and Future Perspectives

This paper described the principal mechanisms of resistance to FLT3 inhibition and the current investigations to overcome it. Secondary on-target mutations (i.e., FLT3-TKD) can be managed by choosing type I inhibitors such as gilteritinib that are consistently active against FLT3-TKD as well as FLT3-ITD, except for a “gatekeeper” F691L mutation. Covalently-coupling FF-10101 and other novel FLT3 inhibitors are now under investigation and have shown promising data on FLT3 F691L. Strategies for secondary off-target abnormalities and a part of primary resistance cannot be simple, regarding the diverse relating genomic abnormalities and complex patterns of clonal evolution. Nevertheless, some genetic abnormalities are/will be clinically targetable, expecting a synergistic anti-tumor effect with FLT3 inhibition. For example, several agents targeting BCL-2, MDM2 and STAT as well as conventional chemotherapy are being evaluated in combination with FLT3 inhibitors. Similarly, abnormal *RAS* and *PIM1* pathways as well as metabolic modifications (e.g., G6PD inactivation) are subject to preclinical investigations. Recent studies have suggested the non-negligible importance of clone-evolutional patterns in terms of acquiring resistance, which possibly affects clinical strategy in managing FLT3-mutated AML. Simply, when you find two distinct targetable mutations and the corresponding agents are available (e.g., FLT3-ITD and *IDH2* mutation), you can choose either one agent if both mutations are limited in a single leukemic clone, but if each mutations are found in different clones, it is worth considering combination or sequential therapy, if allowed. Routine and successive gene sequencing will help detecting additional targetable mutations and determining individual patterns of clone evolution, which would improve our clinical approaches along with the development of novel agents and combination strategies.

Several new agents such as FLT3 inhibitors can create overlapping treatment options, especially in the elderly, unfit AML patients as well as in relapse/refractory AML patients. A lot of clinical trials evaluating the efficacy of promising investigational drugs in AML are ongoing and more drugs will go to the market than ever before. Based on the resistant mechanisms during treatment, how to use these new agents properly is one of the issues with the treatment of AML. Physicians should select an optimal treatment depending on factors such as age, performance status, comorbidities, and genome profiling analysis upon new diagnoses and during treatment.

## Figures and Tables

**Table 1:** FLT3 inhibitors.

Agent		Generation	Type	Selectivity	IC50 (nM)	Drug Sensitivity			
						ITD	D835Y	ITD-D835Y	F691L
Midostaurin	(PKC412)	First	I	Low	139	S	S	R	R
Sunitinib	(SU11248)	First	I	Low	250	S	R	R	R
Lestaurtinib	(CEP701)	First	I	Low	5	S	Int	S	−
Gilteritinib	(ASP2215)	Second	I	Moderate	1.6	S	S	Int	R
Crenolanib	(CP868596)	Second	I	Moderate	2	S	S	Int	R
Sorafenib	(BAY43-9006)	First	II	Moderate	58	S	R	R	R
Tandutinib	(CT53518)	First	II	High	100	S	R	−	−
Quizartinib	(AC220)	Second	II	High	<1.0	S	R	R	R

S (sensitive) means the IC50 is less than or equal to that of FLT3-ITD. R (resistant) means more than two folds increase in IC50. Int (intermediate) remains a 1.0–2.0-folds increase. Here is the reference of selectivity [60,61,62], IC50 for FLT3-ITD [47,49,63,64,65,66,67,68] and drug sensitivity [47,55,69,70,71,72].

**Table 2:** Clinical results of FLT3 inhibitors.

**SORAFENIB (BAY 43-9006)**										
**Authors and Jounals**	**Trial Name**	**Objectives**	**Disease Status**	Agents * Controls Not Shown	**Phase/Design**	**Response Rate**		**Median PFS**	**Median OS**	**Sequential allo-SCT**
Rolling, et al. Lancet Oncol 2015	SORAML	AML (age < 60)	Newly diagnosed	Sorafenib + Standard therapy	II	CR	60% (81/134)	21 mo. [9,10,11,12,13,14,15,16,17,18,19,20,21,22,23,24,25,26,27,28,29,30,31,32]	Not Reached (3-yr OS 63%)	31% (42/132)
Uy, et al. Blood Advances 2016	CALGB 11001	AML (age > 60) with FLT3-ITD and/or TKD	Newly diagnosed	Sorafenib + Standard therapy	II	CR	74% (40/54)	8.8 mo. (FLT3-ITD) 7.8 mo. (FLT3-TKD)	15.0 mo. (FLT3-ITD) 16.2 mo. (FLT3-TKD)	53% (22/54)
Ohanian, et al. Am J Hematol 2018	−	AML (age > 60) with FLT3-ITD	Newly diagnosed	Sorafenib + Azacitidine	I/II	CR/Cri PR	70% (19/27) 7% (2/27)	7.1 mo. (only in responders)	8.3 mo. (in all participants)	11% (3/27)
Sasaki, et al. Cancer 2019	−	AML with FLT3-ITD	Newly diagnosed	Soragenib + Standard therapy	Retrospective	CR/CRi	99% (78/79)	31 mo. [5.7–56.8]	17 mo. [11.1–22.4]	67% (53/79)
Muppidi, et al. Clinical Lymphoma Myeloma and Leukemia 2015	−	AML with FLT3-ITD	Newly diagnosed or relapsed	Sorafenib + Decitabine	Case Series	CR/CRi	83% (5/6)	Not Reported	5.1 mo. [1.9–14.5]	33% (2/6)
Ravandi, et al. Blood 2013	−	AML with FLT3-ITD	Relapsed or refractory (including prior allo-SCT)	Sorafenib + Azacitidine	II	CR/Cri PR	43% (16/37) 3% (1/37)	3.8 mo. [1.0–16.4]	6.2 mo.	16% (6/37)
Bazarbachi, et al. Heamatologica 2019	−	AML with FLT3-ITD	Relapsed ater allo-SCT	Sorafenib as part of/after salvage	Retrospective	CR	39% (10/26)	Not Reported	(2-yr. OS 38%)	13% (3/26)
**MIDOSTAURIN (PKC412)**										
**Authorsand Jounals**	**Trial Name**	**Objectives**	**Disease Status**	Agents * Controls Not Shown	**Phase/Design**	**Response Rate**		**Median PFS**	**Median OS**	**Sequential allo-SCT**
Stone, et al. N Engl J Med 2017	RATIFY	AML with FLT3-ITD and/or TKD	Newly diagnosed	Midostaurin + Standard induction/consolidation	III	CR	70% (504/717)	8.2 mo. [5.4–10.7]	74.7 mo. [31.5–inf.]	57% (287/504)
Schlenk, et al. Blood 2019	AMLSG 16-10	AML with FLT3-ITD and/or TKD	Newly diagnosed	Midostaurin + Standard induction/consolidation f/b Midostaurin maitenance	II	CR/CRi	76% (217/292)	13.2 mo. [10.0–18.3]	26.0 mo. [18.9–37.0]	62% (134/217)
Fischer, et al. J Clin Oncol 2010	−	AML or high-risk MDS	Relapsed or refractory or ineligible to standard therapy	Midostaurin	IIB	PR HI Blast	1% (1/97) 46% (16/35 *) 71% (25/35 *) * only in FLT3-mt	Not Reported	4.3 mo. [3.5–5.2]	31% (42/132)
Strati, et al. Am J Hematol 2015	−	AML or high-risk MDS	Relapsed or refractory or ineligible to standard therapy	Midostaurin + Azacitidine	I/II	CR/Cri PR/MLFS	15% (8/54) 13% (7/54)	4.6 mo. [2.3–6.9] * Duration of Response	5.1 mo. [3.5–6.7]	0% (0/8)
Walker, et al. Leukemia & Lymphoma 2016	−	AML	Relapsed or refractory (including prior allo-SCT)	Midostaurin + Bortezomib + Chemotherapy(MEC)	I	CR/CRi	83% (19/23)	Not Reported	10.8 mo.	63% (12/19)
Maziarz, et al. Blood 2018	RADIUS	AML with FLT3-ITD	in 1st CR after allo-SCT	Midostaurin + Standard of care	II	Not Applicable		(18mo.-PFS 89%)	Not Reported	Not Applicable
**GILTERITINIB (ASP2215)**										
**Authors and Jounals**	**Trial Name**	**Objectives**	**Disease Status**	Agents * Controls Not Shown	**Phase/Design**	**Response Rate**		**Median PFS**	**Median OS**	**Sequential allo-SCT**
Perl, et al. N Engl J Med 2019	ADMIRAL	AML with FLT3-ITD and/or TKD	Relapsed or refractory	Gilteritinib	III	CR/Cri PR	54% (134/247) 13% (33/247)	2.8 mo. [1.4–3.7]	9.3 mo. [7.7–10.7]	26% (63/247)
Perl, et al. Lancet Oncol 2017	−	AML with FLT3-ITD and/or TKD	Relapsed or refractory	Gilteritinib	I/II	CR/Cri PR	41% (69/169) 11% (19/169)	4.6 mo. * Duration of Response	7.1 mo.	22% (37/169)
Usuki, et al. Cancer Science 2018	−	AML	Relapsed or refractory	Gilteritinib	I	CR/Cri PR	60% (3/5 *) 20% (1/5 *) * only in FLT3-mt.	Not Reported	Not Reported	Not Reported
**QUIZARTINIB (AC220)**										
**Authors and Jounals**	**Trial Name**	**Objectives**	**Disease Status**	Agents * Controls Not Shown	**Phase/Design**	**Response Rate**		**Median PFS**	**Median OS**	**Sequential allo-SCT**
Altman, et al. Blood 2018	−	AML	Newly diagnosed	Quizartinib + Standard induction/consolidation f/b Quizartinib maitenance	I	CR/CRi	74% (14/19)	(Maximum 16.3 mo.)	Not Reported	47% (9/19)
Cortes, et al. Blood 2019	QuANTUM-R	AML with FLT3-ITD	Relapsed or refractory	Quizartinib	III	CR/Cri	48% (118/245)	1.4 mo. [0.0–1.9]	6.2 mo. [5.3–7.2]	32% (78/245)
Cortes, et al. Blood 2018	−	AML with FLT3-ITD	Relapsed or refractory	Quizartinib	IIB	CR/Cri PR	47% (36/76) 18% (14/76)	12.3 mo. [9.7–16.1]	22.6 mo. [19.9–28.3]	37% (28/76)
Cortes, et al. Lancet Oncol 2018	−	AML	Relapsed or refractory	Quizartinib	II	CR/Cri PR	50% (125/248) 25% (62/248) * only in ITD-mt.	2.8 mo. [1.4–3.6] * duration of CR, only in ITD-mt.	5.8 mo. [4.9–6.8] * only in ITD-mt.	35% (61/176)
Sandmaier, et al. Am J Hematol 2017	−	AML with FLT3-ITD	in 1st CR after allo-SCT	Quizartinib maintenance	I	Not Applicable		(0.4–22.8 mo.) * duration of maitenance	(3.0–32.7 mo.)	Not Applicable
**CRENOLANIB (CP868596)**										
**Authors and Jounals**	**Trial Name**	**Objectives**	**Disease Status**	Agents * Controls Not Shown	**Phase/Design**	**Response Rate**		**Median PFS**	**Median OS**	**Sequential allo-SCT**
Wang, et al. Blood 2016	−	AML with FLT3-ITD and/or TKD	Newly diagnosed	Crenolanib + Standard induction/consolidation f/b Crenolanib maintenance	II	CR/CRi	96% (24/25)	Not Reported	Not Reached (6 mo. OS 85%)	50% (12/24)
Randhawa, et al. Blood 2014	−	AML with FLT3-ITD and/or TKD	Relapsed or refractory	Crenolanib	II	CR/Cri MLFS HI	23% (3/13) 8% (1/13) 31% (4/13) * only in TKI-naïve	3.0 mo. * only in TKI-naïve	12.7 mo. * only in TKI-naïve	26% (9/34)
Ohanian, et al. Blood 2016	−	AML with FLT3-ITD and/or TKD	Relapsed or refractory	Crenolanib + Salvage chemotherapy (IDA/AraC)	I	CR/CRi	36% (4/11)	Not Reported	8.5 mo.	75% (3/4)
Iyer, et al. Blood 2016	−	AML	Relapsed or refractory	Crenolanib + Chemotherapy (HAM)	I	CR/CRi	67% (4/6)	Not Reported	Not Repoted	25% (1/4)
**LESTAURTINIB (CEP701)**										
**Authors and Jounals**	**Trial Name**	**Objectives**	**Disease Status**	Agents * Controls Not Shown	**Phase/Design**	**Response Rate**		**Median PFS**	**Median OS**	**Sequential allo-SCT**
Levis, et al. Blood 2017	−	AML with FLT3-ITD and/or TKD	Relapsed	Slavage chemotherapy (MEC) f/b Lestaurtinib maintenance	II	CR/CRi	26% (29/112)	Not Reported	5.2 mo.	20% (22/112)
Knapper, et al. Blood 2017	−	AML with FLT3-ITD and/or TKD	Newly diagnosed	Standard induction/consolidation f/b Lestaurtinib maintenance	III	CR/CRi	92% (277/300)	(5-yr. PFS 39–40%)	(5-yr. OS 43–50%)	21% (58/277)

**Table 3:** Clinical trials of FLT3 inhibitors.

**SORAFENIB (BAY 43-9006)**				
**Trial Number**	**Objectives**	**Disease Status**	Agents * Controls Not Shown	**Phase/Design**
NCT01371981	AML with FLT3-ITD (high allelic ratio)	Newly diagnosed	Sorafenib + Bertezomib	III
NCT03170895	AML with FLT3-ITD	Newly diagnosed or relapsed	Sorafenib + Homoharringtonine (STAT inhibitor)	II
**MIDOSTAURIN (PKC412)**				
NCT03686345	Core binding factor AML	Newly diagnosed	Midostaurin + Standard induction	II
NCT03280030	AML with FLT3-ITD and/or TKD	Newly diagnosed	Midostaurin + Standard induction/consolidation f/b Midostaurin maitenance	II
NCT03512197	AML with FLT3-ITD and/or TKD	Newly diagnosed	Midostaurin + Standard induction/consolidation f/b Midostaurin maitenance	III
NCT03379727	AML with FLT3-ITD and/or TKD	Newly diagnosed	Midostaurin + Standard induction/consolidation f/b Midostaurin maitenance	III
**GILTERITINIB (ASP2215)**				
NCT02236013	AML	Newly diagnosed	Gilteritinib + Standard induction/consolidation	I
NCT02752035	AML with FLT3-ITD and/or TKD	Newly diagnosed and ineligible to standard therapy	Gilteritinib + Azacitidine	III
NCT03730012	AML with FLT3-ITD and/or TKD	Relapsed or refractory	Gilteritinib + Atezolizumab (anti-PD-L1 antibody)	I/II
NCT02310321	AML	Newly diagnosed	Gilteritinib + Standard induction/consolidation	I/II
NCT03182244	AML with FLT3-ITD and/or TKD	Relapsed or refractory	Gilteritinib + Salvage chemotherapy	III
**QUIZARTINIB (AC220)**				
NCT02668653	AML with FLT3-ITD	Newly diagnosed	Quizartinib + Standard induction/consolidation	III
NCT03723681	AML	Newly diagnosed	Quizartinib + Standard induction/consolidation	I
NCT02834390	AML	Newly diagnosed	Quizartinib + Standard induction/consolidation	IB
NCT03552029	AML with FLT3-ITD	Relapsed or refractory or ineligible to standard therapy	Quizartinib + Milademetan (MDM2 inhibitor)	I
NCT03135054	AML with FLT3-ITD	Newly diagnosed or relapsed	Quizartinib + Homoharringtonine (STAT inihibitor)	II
NCT03661307	AML with FLT3-ITD	Newly diagnosed or relapsed	Quizartinib + Decitabine + Venetoclax (BCL-2 inhibitor)	I/II
NCT03735875	AML with FLT3-ITD	Relapsed or refractory	Quizartinib + Venetoclax (BCL-2 inhibitor)	IB/II
**CRENOLANIB (CP868596)**				
NCT03258931	AML with FLT3-ITD and/or TKD	Newly diagnosed	Crenolanib + Standard induction/consolidation	III
NCT02283177	AML with FLT3-ITD and/or TKD	Newly diagnosed	Crenolanib + Standard induction	II
NCT03250338	AML with FLT3-ITD and/or TKD	Relapsed or refractory	Crenolanib + Salvage chemotherapy	III
NCT02400281	AML with FLT3-ITD and/or TKD	Relapsed or refractory	Crenolanib + Salvage chemo. or Azacitidine	I/II
NCT02626338	AML	Relapsed or refractory	Crenolanib + Salvage chemotherapy	I/II
NCT01522469	AML with FLT3-ITD and/or TKD	Relapsed or refractory	Crenolanib + Standard induction/consolidation	IB
**OTHERS**				
NCT00783653	AML with FLT3-ITD and/or TKD	Newly diagnosed	Sunitinib + Standard induction	I/II
NCT00469859	AML with FLT3-ITD and/or TKD	Relapsed or refractory	Lestaurtinib + Salvage chemotherapy	I/II

## References

[1] Rosnet O., Mattei M.-G., Marchetto S., Birnbaum D. (1991). Isolation and chromosomal localization of a novel FMS-like tyrosine kinase gene. Genomics.

[2] Rosnet O., Schiff C., Pebusque M.J., Marchetto S., Tonnelle C., Toiron Y., Birg F., Birnbaum D. (1993). Human FLT3/FLK2 gene: cDNA cloning and expression in hematopoietic cells. Blood.

[3] Lemmon M.A., Schlessinger J. (2010). Cell Signaling by Receptor Tyrosine Kinases. Cell.

[4] Small D., Levenstein M., Kim E., Carow C., Amin S., Rockwell P., Witte L., Burrow C., Ratajczak M.Z., Gewirtz A.M. (1994). STK-1, the human homolog of Flk-2/Flt-3, is selectively expressed in CD34+ human bone marrow cells and is involved in the proliferation of early progenitor/stem cells. Proc. Natl. Acad. Sci. USA.

[5] Hannum C., Culpepper J., Campbell D., McClanahan T., Zurawski S., Kastelein R., Bazan J.F., Hudak S., Wagner J., Mattson J. (1994). Ligand for FLT3/FLK2 receptor tyrosine kinase regulates growth of haematopoietic stem cells and is encoded by variant RNAs. Nature.

[6] Lyman S.D., James L., Bos T.V., De Vries P., Brasel K., Gliniak B., Hollingsworth L., Picha K.S., McKenna H.J., Splett R.R. (1993). Molecular cloning of a ligand for the flt3flk-2 tyrosine kinase receptor: A proliferative factor for primitive hematopoietic cells. Cell.

[7] Hayakawa F., Towatari M., Kiyoi H., Tanimoto M., Kitamura T., Saito H., Naoe T. (2000). Tandem-duplicated Flt3 constitutively activates STAT5 and MAP kinase and introduces autonomous cell growth in IL-3-dependent cell lines. Oncogene.

[8] Mizuki M., Fenski R., Halfter H., Matsumura I., Schmidt R., Müller C., Grüning W., Kratz-Albers K., Serve S., Steur C. (2000). Flt3 mutations from patients with acute myeloid leukemia induce transformation of 32D cells mediated by the Ras and STAT5 pathways. Blood.

[9] Meshinchi S., Appelbaum F.R. (2009). Structural and functional alterations of FLT3 in acute myeloid leukemia. Clin. Cancer Res..

[10] Stirewalt D.L., Radich J.P. (2003). The role of FLT3 in haematopoietic malignancies. Nat. Rev. Cancer.

[11] Drexler H.G., Meyer C., Quentmeier H. (1999). Effects of FLT3 Ligand on Proliferation and Survival of Myeloid Leukemia Cells. Leuk. Lymphoma.

[12] Nakao M., Yokota S., Iwai T., Kaneko H., Horiike S., Kashima K., Sonoda Y., Fujimoto T., Misawa S. (1996). Internal tandem duplication of the flt3 gene found in acute myeloid leukemia. Leukemia.

[13] Yamamoto Y. (2001). Activating mutation of D835 within the activation loop of FLT3 in human hematologic malignancies. Blood.

[14] Kiyoi H., Naoe T. (2006). Biology, Clinical Relevance, and Molecularly Targeted Therapy in Acute Leukemia with FLT3 Mutation. Int. J. Hematol..

[15] Kihara R., Nagata Y., Kiyoi H., Kato T., Yamamoto E., Suzuki K., Chen F., Asou N., Ohtake S., Miyawaki S. (2014). Comprehensive analysis of genetic alterations and their prognostic impacts in adult acute myeloid leukemia patients. Leukemia.

[16] Ley T.J., Miller C.A., Ding L., Raphael B.J., Mungall A.J., Robertson A.G., Hoadley K.A., Triche T.J., Laird P.W., Baty J.D. (2013). The Cancer Genome Atlas Research Network; Cancer Genome Atlas Research Network; Genomic and Epigenomic Landscapes of Adult De Novo Acute Myeloid Leukemia. N. Engl. J. Med..

[17] Janke H., Pastore F., Schumacher D., Herold T., Hopfner K.-P., Schneider S., Berdel W.E., Buchner T., Woermann B.J., Subklewe M. (2014). Activating FLT3 Mutants Show Distinct Gain-of-Function Phenotypes In Vitro and a Characteristic Signaling Pathway Profile Associated with Prognosis in Acute Myeloid Leukemia. PLoS ONE.

[18] Okada K., Nogami A., Ishida S., Akiyama H., Chen C., Umezawa Y., Miura O. (2017). FLT3-ITD induces expression of Pim kinases through STAT5 to confer resistance to the PI3K/Akt pathway inhibitors on leukemic cells by enhancing the mTORC1/Mcl-1 pathway. Oncotarget.

[19] Solovey M., Wang Y., Michel C., Metzeler K.H., Herold T., Göthert J.R., Ellenrieder V., Hessmann E., Gattenlöhner S., Neubauer A. (2019). Nuclear factor of activated T-cells, NFATC1, governs FLT3ITD-driven hematopoietic stem cell transformation and a poor prognosis in AML. J. Hematol. Oncol..

[20] Sun Y.-M., Wang W.-T., Zeng Z.-C., Chen T.-Q., Han C., Pan Q., Huang W., Fang K., Sun L.-Y., Zhou Y.-F. (2019). circMYBL2, a circRNA from MYBL2, regulates FLT3 translation by recruiting PTBP1 to promote FLT3-ITD AML progression. Blood.

[21] Zhang Y., Askenazi M., Jiang J., Luckey C.J., Griffin J.D., Marto J.A. (2009). A Robust Error Model for iTRAQ Quantification Reveals Divergent Signaling between Oncogenic FLT3 Mutants in Acute Myeloid Leukemia. Mol. Cell. Proteom..

[22] Klingmüller U., Lorenz U., Cantley L.C., Neel B.G., Lodish H.F. (1995). Specific recruitment of SH-PTP1 to the erythropoietin receptor causes inactivation of JAK2 and termination of proliferative signals. Cell.

[23] Grundler R., Miething C., Thiede C., Peschel C., Duyster J. (2005). FLT3-ITD and tyrosine kinase domain mutants induce 2 distinct phenotypes in a murine bone marrow transplantation model. Blood.

[24] Bailey E., Li L., Duffield A.S., Ma H.S., Huso D.L., Small N. (2013). FLT3/D835Y mutation knock-in mice display less aggressive disease compared with FLT3/internal tandem duplication (ITD) mice. Proc. Natl. Acad. Sci. USA.

[25] Bagrintseva K., Geisenhof S., Kern R., Eichenlaub S., Reindl C., Ellwart J.W., Hiddemann W., Spiekermann K. (2005). FLT3-ITD-TKD dual mutants associated with AML confer resistance to FLT3 PTK inhibitors and cytotoxic agents by overexpression of Bcl-x(L). Blood.

[26] Kiyoi H. (2015). FLT3 Inhibitors: Recent advances and problems for clinical application. Nagoya J. Med. Sci..

[27] Döhner H., Estey E., Grimwade D., Amadori S., Appelbaum F.R., Büchner T., Dombret H., Ebert B.L., Fenaux P., Larson R.A. (2017). Diagnosis and management of AML in adults: 2017 ELN recommendations from an international expert panel. Blood.

[28] Ghiaur G., Levis M.J. (2017). Mechanisms of Resistance to FLT3 Inhibitors and the Role of the Bone Marrow Microenvironment. Hematol. Clin. N. Am..

[29] Zhou J., Chng W.-J. (2018). Resistance to FLT3 inhibitors in acute myeloid leukemia: Molecular mechanisms and resensitizing strategies. World J. Clin. Oncol..

[30] Daver N., Schlenk R.F., Russell N.H., Levis M.J. (2019). Targeting FLT3 mutations in AML: Review of current knowledge and evidence. Leukemia.

[31] Port M., Böttcher M., Thol F., Ganser A., Schlenk R., Wasem J., Neumann A., Pouryamout L. (2014). Prognostic significance of FLT3 internal tandem duplication, nucleophosmin 1, and CEBPA gene mutations for acute myeloid leukemia patients with normal karyotype and younger than 60 years: A systematic review and meta-analysis. Ann. Hematol..

[32] Thiede C., Steudel C., Mohr B., Schaich M., Schäkel U., Platzbecker U., Wermke M., Bornhäuser M., Ritter M., Neubauer A. (2002). Analysis of FLT3-activating mutations in 979 patients with acute myelogenous leukemia: Association with FAB subtypes and identification of subgroups with poor prognosis. Blood.

[33] Schlenk R.F., Kayser S., Bullinger L., Kobbe G., Casper J., Ringhoffer M., Held G., Brossart P., Lübbert M., Salih H.R. (2014). Differential impact of allelic ratio and insertion site in FLT3-ITD–positive AML with respect to allogeneic transplantation. Blood.

[34] Yalniz F.F., Dalle I.A., Kantarjian H.M., Borthakur G., Kadia T.M., Patel K., Loghavi S., Garcia-Manero G., Sasaki K., Daver N. (2019). Prognostic significance of baselineFLT3-ITD mutant allele level in acute myeloid leukemia treated with intensive chemotherapy with/without sorafenib. Am. J. Hematol..

[35] Pratcorona M., Brunet S., Nomdedéu J.F., Ribera J., Tormo M., Duarte R., Escoda L., Guàrdia R., De Llano M.P.Q., Salamero O. (2013). Favorable outcome of patients with acute myeloid leukemia harboring a low-allelic burden FLT3-ITD mutation and concomitant NPM1 mutation: Relevance to post-remission therapy. Blood.

[36] Linch D.C., Hills R.K., Burnett A.K., Khwaja A., Gale R.E. (2014). Impact of FLT3ITD mutant allele level on relapse risk in intermediate-risk acute myeloid leukemia. Blood.

[37] Versluis J., Devillier R., Van Putten W.L.J., Manz M.G., Vekemans M.-C., Legdeur M.-C., Passweg J.R., Maertens J., Kuball J., Biemond B.J. (2016). Comparative value of post-remission treatment in cytogenetically normal AML subclassified by NPM1 and FLT3-ITD allelic ratio. Leukemia.

[38] Sakaguchi M., Yamaguchi H., Najima Y., Usuki K., Ueki T., Oh I., Mori S., Kawata E., Uoshima N., Kobayashi Y. (2018). Prognostic impact of low allelic ratio FLT3-ITD and NPM1 mutation in acute myeloid leukemia. Blood Adv..

[39] Bacher U., Haferlach C., Kern W., Haferlach T., Schnittger S. (2008). Prognostic relevance of FLT3-TKD mutations in AML: The combination matters—An analysis of 3082 patients. Blood.

[40] Zhang H., Savage S., Reister-Schultz A., Bottomly D., White L., Segerdell E., Wilmot B., McWeeney S.K., Eide C.A., Nechiporuk T. (2019). Clinical resistance to crenolanib in acute myeloid leukemia due to diverse molecular mechanisms. Nat. Commun..

[41] Kelly L.M., Yu J.-C., Boulton C.L., Apatira M., Li J., Sullivan C.M., Williams I., Amaral S.M., Curley D.P., Duclos N. (2002). CT53518, a novel selective FLT3 antagonist for the treatment of acute myelogenous leukemia (AML). Cancer Cell.

[42] Smith B.D., Levis M., Beran M., Giles F., Kantarjian H., Berg K., Murphy K.M., Dauses T., Allebach J., Small D. (2004). Single-agent CEP-701, a novel FLT3 inhibitor, shows biologic and clinical activity in patients with relapsed or refractory acute myeloid leukemia. Blood.

[43] Stone R.M., DeAngelo D.J., Klimek V., Galinsky I., Estey E., Nimer S.D., Grandin W., Lebwohl D., Wang Y., Cohen P. (2005). Patients with acute myeloid leukemia and an activating mutation in FLT3 respond to a small-molecule FLT3 tyrosine kinase inhibitor, PKC412. Blood.

[44] Fiedler W., Serve H., Döhner H., Schwittay M., Ottmann O.G., O’Farrell A.-M., Bello C.L., Allred R., Manning W.C., Cherrington J.M. (2005). A phase 1 study of SU11248 in the treatment of patients with refractory or resistant acute myeloid leukemia (AML) or not amenable to conventional therapy for the disease. Blood.

[45] Ravandi F., Cortes J.E., Jones D., Faderl S., Garcia-Manero G., Konopleva M.Y., O’Brien S., Estrov Z., Borthakur G., Thomas D. (2010). Phase I/II Study of Combination Therapy with Sorafenib, Idarubicin, and Cytarabine in Younger Patients with Acute Myeloid Leukemia. J. Clin. Oncol..

[46] Mori M., Kaneko N., Ueno Y., Yamada M., Tanaka R., Saito R., Shimada I., Mori K., Kuromitsu S. (2017). Gilteritinib, a FLT3/AXL inhibitor, shows antileukemic activity in mouse models of FLT3 mutated acute myeloid leukemia. Investig. New Drugs.

[47] Lee L.Y., Hernandez D., Rajkhowa T., Smith S.C., Raman J., Nguyen B., Small N., Levis M. (2017). Preclinical studies of gilteritinib, a next-generation FLT3 inhibitor. Blood.

[48] Zarrinkar P.P., Gunawardane R.N., Cramer M.D., Gardner M.F., Brigham D., Belli B., Karaman M.W., Pratz K.W., Pallares G., Chao Q. (2009). AC220 is a uniquely potent and selective inhibitor of FLT3 for the treatment of acute myeloid leukemia (AML). Blood.

[49] Galanis A., Ma H., Rajkhowa T., Ramachandran A., Small N., Cortes J., Levis M. (2014). Crenolanib is a potent inhibitor of FLT3 with activity against resistance-conferring point mutants. Blood.

[50] Smith C.C., Lasater E.A., Lin K.C., Wang Q., McCreery M.Q., Stewart W.K., Damon L.E., Perl A.E., Jeschke G., Sugita M. (2014). Crenolanib is a selective type I pan-FLT3 inhibitor. Proc. Natl. Acad. Sci. USA.

[51] Angiolini M. (2011). Targeting the DFG-in kinase conformation: A new trend emerging from a patent analysis. Future Med. Chem..

[52] Ke Y.-Y., Singh V.K., Coumar M.S., Hsu Y.C., Wang W.-C., Song J.-S., Chen C.-H., Lin W.-H., Wu S.-H., Hsu J.T.A. (2015). Homology modeling of DFG-in FMS-like tyrosine kinase 3 (FLT3) and structure-based virtual screening for inhibitor identification. Sci. Rep..

[53] Bhullar K.S., Lagarón N.O., McGowan E.M., Parmar I., Jha A., Hubbard B.P., Rupasinghe H.P.V. (2018). Kinase-targeted cancer therapies: Progress, challenges and future directions. Mol. Cancer.

[54] Kiyoi H., Kawashima N., Ishikawa Y. (2019). FLT3 mutations in acute myeloid leukemia: Therapeutic paradigm beyond inhibitor development. Cancer Sci..

[55] Smith C.C., Lin K., Stecula A., Sali A., Shah N.P. (2015). FLT3 D835 mutations confer differential resistance to type II FLT3 inhibitors. Leukemia.

[56] Williams A.B., Nguyen B., Li L., Brown P., Levis M., Leahy D., Small N. (2012). Mutations of FLT3/ITD confer resistance to multiple tyrosine kinase inhibitors. Leukemia.

[57] Cools J. (2004). Prediction of Resistance to Small Molecule FLT3 Inhibitors: Implications for Molecularly Targeted Therapy of Acute Leukemia. Cancer Res..

[58] McMahon C.M., Canaani J., Rea B., McMahon C.M., Canaani J., Rea B., Sargent R.L., Morrissette J.J., Lieberman D.B., Watt C. (2017). Mechanisms of Acquired Resistance to Gilteritinib Therapy in Relapsed and Refractory FLT3 -Mutated Acute Myeloid Leukemia. Blood.

[59] Staudt D., Murray H.C., McLachlan T., Alvaro F., Enjeti A.K., Verrills N.M., Dun M.D. (2018). Targeting Oncogenic Signaling in Mutant FLT3 Acute Myeloid Leukemia: The Path to Least Resistance. Int. J. Mol. Sci..

[60] Eid S., Turk S., Volkamer A., Rippmann F., Fulle S. (2017). KinMap: A web-based tool for interactive navigation through human kinome data. BMC Bioinform..

[61] Klaeger S., Heinzlmeir S., Wilhelm M., Polzer H., Vick B., Koenig P.-A., Reinecke M., Ruprecht B., Wiechmann S., Meng C. (2017). The target landscape of clinical kinase drugs. Science.

[62] Karaman M.W., Herrgard S., Treiber D.K., Gallant P., Atteridge C.E., Campbell B.T., Chan K.W., Ciceri P., Davis M.I., Edeen P.T. (2008). A quantitative analysis of kinase inhibitor selectivity. Nat. Biotechnol..

[63] Weisberg E., Puissant A., Stone R., Sattler M., Buhrlage S.J., Yang J., Manley P.W., Meng C., Buonopane M., Daley J.F. (2017). Characterization of midostaurin as a dual inhibitor of FLT3 and SYK and potentiation of FLT3 inhibition against FLT3-ITD-driven leukemia harboring activated SYK kinase. Oncotarget.

[64] O’Farrell A.-M., Abrams T.J., Yuen H.A., Ngai T.J., Louie S.G., Yee K.W.H., Wong L.M., Hong W., Lee L.B., Town A. (2003). SU11248 is a novel FLT3 tyrosine kinase inhibitor with potent activity in vitro and in vivo. Blood.

[65] Levis M.J., Allebach J., Tse K.-F., Zheng R., Baldwin B.R., Smith B.D., Jones-Bolin S., Ruggeri B., Dionne C., Small N. (2002). A FLT3-targeted tyrosine kinase inhibitor is cytotoxic to leukemia cells in vitro and in vivo. Blood.

[66] Mori S., Cortes J., Kantarjian H., Zhang W., Andreef M., Ravandi F. (2008). Potential role of sorafenib in the treatment of acute myeloid leukemia. Leuk. Lymphoma.

[67] Schittenhelm M.M., Kampa K.M., Yee K.W.H., Heinrich M.C. (2009). The FLT3 inhibitor tandutinib (formerly MLN518) has sequence-independent synergistic effects with cytarabine and daunorubicin. Cell Cycle.

[68] Aikawa T., Togashi N., Iwanaga K., Okada H., Nishiya Y., Inoue S., Levis M.J., Isoyama T. (2020). Quizartinib, a selective FLT3 inhibitor, maintains antileukemic activity in preclinical models of RAS-mediated midostaurin-resistant acute myeloid leukemia cells. Oncotarget.

[69] Nguyen B., Williams A.B., Young D.J., Ma H., Li L., Levis M., Brown P., Small N. (2017). FLT3 activating mutations display differential sensitivity to multiple tyrosine kinase inhibitors. Oncotarget.

[70] Barry E.V., Clark J.J., Cools J., Roesel J., Gilliland D.G. (2007). Uniform sensitivity of FLT3 activation loop mutants to the tyrosine kinase inhibitor midostaurin. Blood.

[71] Yee K.W.H., Schittenhelm M., O’Farrell A.-M., Town A.R., McGreevey L., Bainbridge T., Cherrington J.M., Heinrich M.C. (2004). Synergistic effect of SU11248 with cytarabine or daunorubicin on FLT3 ITD–positive leukemic cells. Blood.

[72] Schittenhelm M.M., Heinrich M.C., Akmut F., Döhner H., Döhner K., Schittenhelm M.M. (2013). Quizartinib (AC220) is a potent second generation class III tyrosine kinase inhibitor that displays a distinct inhibition profile against mutant-FLT3, -PDGFRA and -KIT isoforms. Mol. Cancer.

[73] Stone R.M., Mandrekar S.J., Sanford B.L., Laumann K., Geyer S., Bloomfield C.D., Thiede C., Prior T.W., Döhner K., Marcucci G. (2017). Midostaurin plus Chemotherapy for Acute Myeloid Leukemia with a FLT3 Mutation. N. Engl. J. Med..

[74] Schlenk R.F., Weber D., Fiedler W., Salih H.R., Wulf G., Salwender H., Schroeder T., Kindler T., Lübbert M., Wolf D. (2019). Midostaurin added to chemotherapy and continued single-agent maintenance therapy in acute myeloid leukemia with FLT3-ITD. Blood.

[75] Perl A.E., Martinelli G., Cortes J.E., Neubauer A., Berman E., Paolini S., Montesinos P., Baer M.R., Larson R.A., Ustun C. (2019). Gilteritinib or Chemotherapy for Relapsed or Refractory FLT3-Mutated AML. N. Engl. J. Med..

[76] Cortes J., Khaled S., Martinelli G., Perl A.E., Ganguly S., Russell N., Krämer A., Dombret H., Hogge D., Jonas B.A. (2019). Quizartinib versus salvage chemotherapy in relapsed or refractory FLT3-ITD acute myeloid leukaemia (QuANTUM-R): A multicentre, randomised, controlled, open-label, phase 3 trial. Lancet Oncol..

[77] Altman J.K., Foran J.M., Pratz K.W., Trone D., Cortes J.E., Tallman M.S. (2017). Phase 1 study of quizartinib in combination with induction and consolidation chemotherapy in patients with newly diagnosed acute myeloid leukemia. Am. J. Hematol..

[78] Röllig C., Serve H., Hüttmann A., Noppeney R., Mueller-Tidow C., Krug U., Baldus C.D., Brandts C.H., Kunzmann V., Einsele H. (2015). Addition of sorafenib versus placebo to standard therapy in patients aged 60 years or younger with newly diagnosed acute myeloid leukaemia (SORAML): A multicentre, phase 2, randomised controlled trial. Lancet Oncol..

[79] Uy G.L., Mandrekar S.J., Laumann K., Marcucci G., Zhao W., Levis M.J., Klepin H.D., Baer M.R., Powell B.L., Westervelt P. (2017). A phase 2 study incorporating sorafenib into the chemotherapy for older adults with FLT3-mutated acute myeloid leukemia: CALGB 11001. Blood Adv..

[80] Ohanian M., Garcia-Manero G., Levis M.J., Jabbour E., Daver N., Borthakur G., Kadia T., Pierce S., Burger J., Richie M.A. (2018). Sorafenib Combined with 5-azacytidine in Older Patients with Untreated FLT3-ITD Mutated Acute Myeloid Leukemia. Am. J. Hematol..

[81] Sasaki K., Kantarjian H.M., Kadia T.M., Patel K., Loghavi S., Garcia-Manero G., Jabbour E.J., Dinardo C.D., Pemmaraju N., Daver N. (2019). Sorafenib plus intensive chemotherapy improves survival in patients with newly diagnosed, FLT3-internal tandem duplication mutation–positive acute myeloid leukemia. Cancer.

[82] Muppidi M.R., Portwood S., Griffiths E.A., Thompson J.E., Ford L.A., Freyer C.W., Wetzler M., Wang E.S. (2015). Decitabine and Sorafenib Therapy in FLT-3 ITD-Mutant Acute Myeloid Leukemia. Clin. Lymphoma Myeloma Leuk..

[83] Ravandi F., Alattar M.L., Grunwald M.R., Rudek M.A., Rajkhowa T., Richie M.A., Pierce S., Daver N., Garcia-Manero G., Faderl S. (2013). Phase 2 study of azacytidine plus sorafenib in patients with acute myeloid leukemia and FLT-3 internal tandem duplication mutation. Blood.

[84] Bazarbachi A., Labopin M., Battipaglia G., Djabali A., Passweg J., Socié G., Forcade E., Blaise D., Chevallier P., Orvain C. (2019). Sorafenib improves survival of FLT3-mutated acute myeloid leukemia in relapse after allogeneic stem cell transplantation: A report of the EBMT Acute Leukemia Working Party. Haematologica.

[85] Wang E.S., Stone R.M., Tallman M.S., Walter R.B., Eckardt J.R., Collins R. (2016). Crenolanib, a Type I FLT3 TKI, Can be Safely Combined with Cytarabine and Anthracycline Induction Chemotherapy and Results in High Response Rates in Patients with Newly Diagnosed FLT3 Mutant Acute Myeloid Leukemia (AML). Blood.

[86] Iyer S.P., Jethava Y., Karanes C., Eckardt J.R., Collins R. (2016). Safety Study of Salvage Chemotherapy High-Dose Ara-C/Mitoxantrone (HAM) and Type I FLT3-TKI Crenolanib in First Relapsed/Primary Refractory AML. Blood.

[87] Ohanian M., Kantarjian H.M., Borthakur G., Kadia T.M., Konopleva M., Garcia-Manero G., Estrov Z., Ferrajoli A., Takahashi K., Jabbour E.J. (2016). Efficacy of a Type I FLT3 Inhibitor, Crenolanib, with Idarubicin and High-Dose Ara-C in Multiply Relapsed/Refractory FLT3+ AML. Blood.

[88] Levis M.J., Ravandi F., Wang E.S., Baer M.R., Perl A., Coutre S., Erba H., Stuart R.K., Baccarani M., Cripe L.D. (2011). Results from a randomized trial of salvage chemotherapy followed by lestaurtinib for patients with FLT3 mutant AML in first relapse. Blood.

[89] Knapper S., Russell N., Gilkes A., Hills R.K., Gale R.E., Cavenagh J.D., Jones G., Kjeldsen L., Grunwald M.R., Thomas I. (2017). A randomized assessment of adding the kinase inhibitor lestaurtinib to first-line chemotherapy for FLT3-mutated AML. Blood.

[90] Sato T., Yang X., Knapper S., White P., Smith B.D., Galkin S., Small D., Burnett A., Levis M.J. (2011). FLT3 ligand impedes the efficacy of FLT3 inhibitors in vitro and in vivo. Blood.

[91] Chen F., Ishikawa Y., Akashi A., Naoe T., Kiyoi H. (2016). Co-expression of wild-type FLT3 attenuates the inhibitory effect of FLT3 inhibitor on FLT3 mutated leukemia cells. Oncotarget.

[92] Traer E., Martinez J., Javidi-Sharifi N., Agarwal A., Dunlap J., English I., Kovacsovics T., Tyner J.W., Wong M., Druker B.J. (2016). FGF2 from Marrow Microenvironment Promotes Resistance to FLT3 Inhibitors in Acute Myeloid Leukemia. Cancer Res..

[93] Jacobi A., Thieme S., Lehmann R., Ugarte F., Malech H.L., Koch S., Thiede C., Müller K., Bornhäuser M., Ryser M. (2009). Impact of CXCR4 inhibition on FLT3-ITD-positive human AML blasts. Exp. Hematol..

[94] Zeng Z., Shi Y.X., Samudio I.J., Wang R.-Y., Ling X., Frolova O., Levis M., Rubin J.B., Negrin R.R., Estey E.H. (2009). Targeting the leukemia microenvironment by CXCR4 inhibition overcomes resistance to kinase inhibitors and chemotherapy in AML. Blood.

[95] Chang Y.-T., Hernandez D., Alonso S., Gao M., Su M., Ghiaur G., Levis M.J., Jones R.J. (2019). Role of CYP3A4 in bone marrow microenvironment–mediated protection of FLT3/ITD AML from tyrosine kinase inhibitors. Blood Adv..

[96] Smith C.C., Wang Q., Chin C.-S., Salerno S., Damon L.E., Levis M.J., Perl A.E., Travers K.J., Wang S., Hunt J.P. (2012). Validation of ITD mutations in FLT3 as a therapeutic target in human acute myeloid leukaemia. Nature.

[97] McMahon C.M., Ferng T., Canaani J., Wang E.S., Morrissette J.J., Eastburn D.J., Pellegrino M., Durruthy-Durruthy R., Watt C.D., Asthana S. (2019). Clonal Selection with RAS Pathway Activation Mediates Secondary Clinical Resistance to Selective FLT3 Inhibition in Acute Myeloid Leukemia. Cancer Discov..

[98] Piloto O., Wright M., Brown P., Kim K.-T., Levis M., Small D. (2007). Prolonged exposure to FLT3 inhibitors leads to resistance via activation of parallel signaling pathways. Blood.

[99] Park I.-K., Mundy-Bosse B., Whitman S.P., Zhang X., Warner S.L., Bearss D., Blum W., Marcucci G., Caligiuri M.A. (2015). Receptor tyrosine kinase Axl is required for resistance of leukemic cells to FLT3-targeted therapy in acute myeloid leukemia. Leukemia.

[100] Dumas P.-Y., Naudin C., Martin-Lannerée S., Izac B., Casetti L., Mansier O., Rousseau B., Artus A., Dufossée M., Giese A. (2019). Hematopoietic niche drives FLT3-ITD acute myeloid leukemia resistance to quizartinib via STAT5-and hypoxia-dependent upregulation of AXL. Haematologica.

[101] Dinardo C.D., Stein E.M., De Botton S., Roboz G.J., Altman J.K., Mims A.S., Swords R., Collins R.H., Mannis G.N., Pollyea D.A. (2018). Durable Remissions with Ivosidenib inIDH1-Mutated Relapsed or Refractory AML. N. Engl. J. Med..

[102] Stein E.M., Dinardo C.D., Fathi A.T., Pollyea D.A., Stone R.M., Altman J.K., Roboz G.J., Patel M.R., Collins R., Flinn I.W. (2019). Molecular remission and response patterns in patients with mutant-IDH2 acute myeloid leukemia treated with enasidenib. Blood.

[103] Stein E.M., Dinardo C.D., Pollyea D.A., Fathi A.T., Roboz G.J., Altman J.K., Stone R.M., DeAngelo D.J., Levine R.L., Flinn I.W. (2017). Enasidenib in mutant IDH2 relapsed or refractory acute myeloid leukemia. Blood.

[104] Lindblad O., Cordero E., Puissant A., Macaulay L., Ramos A., Kabir N.N., Sun J., Vallon-Christersson J., Haraldsson K., Hemann M.T. (2016). Aberrant activation of the PI3K/mTOR pathway promotes resistance to sorafenib in AML. Oncogene.

[105] Tursynbay Y., Zhang J., Li Z., Tokay T., Zhumadilov Z., Wu D., Xie Y. (2015). Pim-1 kinase as cancer drug target: An update. Biomed. Rep..

[106] Kim K.-T., Baird K., Ahn J.-Y., Meltzer P., Lilly M., Levis M.J., Small N. (2005). Pim-1 is up-regulated by constitutively activated FLT3 and plays a role in FLT3-mediated cell survival. Blood.

[107] Green A.S., Maciel T., Hospital M.-A., Yin C., Mazed F., Townsend E.C., Pilorge S., Lambert M., Paubelle E., Jacquel A. (2015). Pim kinases modulate resistance to FLT3 tyrosine kinase inhibitors in FLT3-ITD acute myeloid leukemia. Sci. Adv..

[108] Kohl T.M., Hellinger C., Ahmed F., Buske C., Hiddemann W., Bohlander S.K., Spiekermann K. (2007). BH3 mimetic ABT-737 neutralizes resistance to FLT3 inhibitor treatment mediated by FLT3-independent expression of BCL2 in primary AML blasts. Leukemia.

[109] Breitenbuecher F., Markova B., Kasper S., Carius B., Stauder T., Böhmer F.D., Masson K., Rönnstrand L., Huber C., Kindler T. (2009). A novel molecular mechanism of primary resistance to FLT3-kinase inhibitors in AML. Blood.

[110] Yoshimoto G., Miyamoto T., Jabbarzadeh-Tabrizi S., Iino T., Rocnik J.L., Kikushige Y., Mori Y., Shima T., Iwasaki H., Takenaka K. (2009). FLT3-ITD up-regulates MCL-1 to promote survival of stem cells in acute myeloid leukemia via FLT3-ITD–specific STAT5 activation. Blood.

[111] Yamaura T., Nakatani T., Uda K., Ogura H., Shin W., Kurokawa N., Saito K., Fujikawa N., Date T., Takasaki M. (2018). A novel irreversible FLT3 inhibitor, FF-10101, shows excellent efficacy against AML cells with FLT3 mutations. Blood.

[112] Czardybon W., Windak R., Gołas A., Gałezowski M., Sabiniarz A., Dolata I., Salwińska M., Guzik P., Zawadzka M., Gabor-Worwa E. (2018). A novel, dual pan-PIM/FLT3 inhibitor SEL24 exhibits broad therapeutic potential in acute myeloid leukemia. Oncotarget.

[113] Xu B., Zhao Y., Wang X., Gong P., Ge W. (2016). MZH29 is a novel potent inhibitor that overcomes drug resistance FLT3 mutations in acute myeloid leukemia. Leukemia.

[114] Minson K.A., Smith C.C., DeRyckere D., Libbrecht C., Sherick A.L., Huey M.G., Lasater E.A., Kirkpatrick G.D., Stashko M.A., Zhang W. (2016). The MERTK/FLT3 inhibitor MRX-2843 overcomes resistance-conferring FLT3 mutations in acute myeloid leukemia. JCI Insight.

[115] Zirm E., Spies-Weisshart B., Heidel F.H., Schnetzke U., Böhmer F.-D., Hochhaus A., Fischer T., Scholl S. (2012). Ponatinib may overcome resistance of FLT3-ITD harbouring additional point mutations, notably the previously refractory F691I mutation. Br. J. Haematol..

[116] Sung L., Blonquist T.M., Hernandez D., Amrein P.C., Ballen K.K., McMasters M., Avigan D., Joyce R., Logan E.K., Hobbs G.S. (2017). Cabozantinib is well tolerated in acute myeloid leukemia and effectively inhibits the resistance-conferring FLT3/tyrosine kinase domain/F691 mutation. Cancer.

[117] Wang A., Wu H., Chen C., Hu C., Qi Z., Wang W., Yu K., Liu X., Zou F., Zhao Z. (2016). Dual inhibition of AKT/FLT3-ITD by A674563 overcomes FLT3 ligand-induced drug resistance in FLT3-ITD positive AML. Oncotarget.

[118] Zhang W., Borthakur G., Gao C., Chen Y., Mu H., Ruvolo V.R., Nomoto K., Zhao N., Konopleva M., Andreeff M. (2016). The Dual MEK/FLT3 Inhibitor E6201 Exerts Cytotoxic Activity against Acute Myeloid Leukemia Cells Harboring Resistance-Conferring FLT3 Mutations. Cancer Res..

[119] Larrue C., Saland E., Boutzen H., Vergez F., David M., Joffre C., Hospital M.-A., Tamburini J., Delabesse E., Manenti S. (2016). Proteasome inhibitors induce FLT3-ITD degradation through autophagy in AML cells. Blood.

[120] Nagai K., Hou L., Li L., Nguyen B., Seale T., Shirley C., Ma H., Levis M., Ghiaur G., Duffield A. (2018). Combination of ATO with FLT3 TKIs eliminates FLT3/ITD+ leukemia cells through reduced expression of FLT3. Oncotarget.

[121] Uras I.Z., Walter G.J., Scheicher R., Bellutti F., Prchal-Murphy M., Tigan A.S., Valent P., Heidel F.H., Kubicek S., Scholl C. (2016). Palbociclib treatment of FLT3-ITD+ AML cells uncovers a kinase-dependent transcriptional regulation of FLT3 and PIM1 by CDK6. Blood.

[122] Gregory M., D’Alessandro A., Alvarez-Calderon F., Kim J., Nemkov T., Adane B., Rozhok A.I., Kumar A., Kumar V., Pollyea D.A. (2016). ATM/G6PD-driven redox metabolism promotes FLT3 inhibitor resistance in acute myeloid leukemia. Proc. Natl. Acad. Sci. USA.

[123] Dickson G.J., Bustraan S., Hills R.K., Ali A., Goldstone A.H., Burnett A.K., Linch D.C., Gale R.E. (2015). The value of molecular stratification for CEBPA DM and NPM1 MUT FLT3 WT genotypes in older patients with acute myeloid leukaemia. Br. J. Haematol..

[124] Rothenberg-Thurley M., Amler S., Goerlich D., Kohnke T., Konstandin N.P., Schneider S., Sauerland M.C., Herold T., Hubmann M., Ksienzyk B. (2018). Persistence of pre-leukemic clones during first remission and risk of relapse in acute myeloid leukemia. Leukemia.

[125] Canaani J., Labopin M., Huang X.-J., Arcese W., Ciceri F., Blaise D., Irrera G., Corral L.L., Bruno B., Santarone S. (2018). T-cell replete haploidentical stem cell transplantation attenuates the prognostic impact of FLT3-ITD in acute myeloid leukemia: A report from the Acute Leukemia Working Party of the European Society for Blood and Marrow Transplantation. Am. J. Hematol..

[126] Hou P., Wu C., Wang Y., Qi R., Bhavanasi D., Zuo Z., Dos Santos C., Chen S., Chen Y., Zheng H. (2017). A Genome-Wide CRISPR Screen Identifies Genes Critical for Resistance to FLT3 Inhibitor AC220. Cancer Res..

[127] Miyamoto K., Minami Y. (2019). Precision medicine and novel molecular target therapies in acute myeloid leukemia: The background of hematologic malignancies (HM)-SCREEN-Japan 01. Int. J. Clin. Oncol..

